# Effects of anabolic steroids on acute phase responses in intra-abdominal sepsis

**DOI:** 10.1080/09629359791965

**Published:** 1997-02

**Authors:** K. Mealy, A. Clooney, P. Marks, T. Hennessy, J. Jackson

**Affiliations:** 1 Department of Surgery Trinity College and St James's Hospital Dublin Ireland; 2 Department of Immunology St James's Hospital and Dublin Institute of Technology Dublin Ireland; 3 Department of Surgery Western General Hospital Crewe Road Edinburgh EH4 2XU UK

## Abstract

The acute phase response is an important adaptive response to sepsis and injury. As anabolic steroids increase protein synthesis we postulated that these agents might also increase hepatic acute phase protein synthesis. Male Wistar rats were pretreated with testosterone or danazol for 48 h prior to caecal ligation and puncture (CLP). Thirty-six h following surgery the animals were killed and blood taken for full blood count, total protein, albumin, α, β and γ globulin fractions on serum electrophoresis, complement C_3_ and transferrin levels. Danazol increased the α1, α2 and β1 globulin serum protein fractions in comparison with no surgery and CLP alone groups. These results indicate that danazol increases plasma acute phase proteins, as measured by electrophoresis, in this model of intra-abdominal sepsis.


Mediators of Inflammation, **6**, 69–72 (1997)

### Effects of anabolic steroids on acute phase responses in intra-abdominal sepsis

K. Mealy,[1,CA*] A. Clooney,[2] P. Marks,[1] T. Hennessy[1] and J. Jackson[2]

[1]Department of Surgery, Trinity College and St James's Hospital, Dublin, Ireland; and [3]Department of Immunology, St James's Hospital and Dublin Institute of Technology, Dublin, Ireland
*Department of Surgery, Western General Hospital, Crewe Road, Edinburgh EH4 2XU, UK

[CA]*Corresponding Author and Address
Fax: (+44) 131 343 6529

**THE acute phase response is an important adaptive response to sepsis and injury. As anabolic steroids increase protein synthesis we postulated that these agents might also increase hepatic acute phase protein synthesis. Male Wistar rats were pretreated with testosterone or danazol for 48 h prior to caecal ligation and puncture (CLP). Thirty-six h following surgery the animals were killed and blood taken for full blood count, total protein, albumin, α, β and γ globulin fractions on serum electrophoresis, complement $C_3$ and transferrin levels. Danazol increased the $\alpha_1$, $\alpha_2$ and $\beta_1$ globulin serum protein fractions in comparison with no surgery and CLP alone groups. These results indicate that danazol increases plasma acute phase proteins, as measured by electrophoresis, in this model of intra-abdominal sepsis.**

**Key words:** Acute phase response, Anabolic steroids, Intra-abdominal sepsis

### Introduction

The acute phase response refers to a series of physiological changes in response to inflammation which include pyrexia, anorexia and increased production of plasma acute phase proteins which are mainly of hepatic origin.[1] These proteins include anti-proteases which limit tissue destruction, and metal binding proteins which are thought to inhibit bacterial multiplication. In humans, the major positive acute phase protein is C-reactive protein which acts as an opsonin and helps in the phagocytosis of foreign particles. Some hepatic acute phase proteins such as albumin decrease during inflammation and are referred to as negative acute phase proteins. In rats, the major positive acute phase proteins are $\alpha_1$-glycoprotein and $\alpha_2$-macroglobulin while $\alpha_1$ inhibitor 3 is the major negative acute phase protein.[2]

The importance of the acute phase response is highlighted by its persistence during evolutionary history. Sepsis induces a consistent hepatic acute phase response and in severe sepsis an adequate hepatic acute phase response may be important for survival,[3] therefore manipulation of the acute phase response might be of benefit in improving outcome in sepsis. This has been shown in the treatment of fulminant sepsis where the use of recombinant forms of protease inhibitors such as antithrombin III is an important advance in the therapy of this disorder.[4,5] As major surgery can also be a trigger for organ failure and life-threatening sepsis it is possible that augmentation of acute phase responses prior to surgery may be of benefit in surgical patients.

In these studies we examined the role of the androgenic steroids testosterone and danazol, which are known to increase skeletal muscle protein synthesis, in augmenting hepatic acute phase proteins in an animal model of intra-abdominal sepsis.

### Materials and Methods

Male Wistar rats were randomized into five groups. Three groups underwent caecal ligation and puncture (CLP) and a fourth group underwent sham operation ($n = 12$): all under neuroleptic anaesthesia (ketamine/xylazine). A CLP alone group received CLP and no treatment ($n = 9$) and the two other CLP groups received either testosterone (Test, $n = 12$, 2.5 mg testosterone propionate) or danazol (Dan, $n = 12$, 20 mg/kg/day). Post-operative analgesia was administered as necessary by subcutaneous injection (buprenorphine hydrocloride). The testosterone was administered as a single depot injection 48 h prior to surgery and the danazol was given by gavage, twice daily for 48 h before surgery and following surgery until the end of

© 1997 Rapid Science Publishers the study period. The animals were killed 36 h following surgery as in our laboratory this CLP model has a mortality of approximately 20% at 48 h. All animals were allowed rat chow and water *ad libitum*. The fifth group underwent no surgery or treatment ($n = 6$).

The animals were killed by $CO_2$ inhalation and exsanguinated by cardiac puncture. Blood was taken into EDTA bottles for full blood count and serum was also obtained for total protein estimation and serum electrophoresis. Full blood counts were performed on a Coulter STKS Automated Haematology Analyzer and differential white cell counts performed manually following Giemsa staining of blood films. Total serum protein was measured by refractometry and albumin, $\alpha$, $\beta$ and $\gamma$ globulin fractions were measured by electrophoresis followed by scanning densitometry. The $\alpha$ and $\beta$ globulin fractions were subdivided into $\alpha_1$ and $\alpha_2$ and $\beta_1$ and $\beta_2$ groups for quantification. Transferrin and complement $C_3$ levels were measured by laser nephalometry.

### Results

### Survival

There were no treatment associated deaths during the 36 h study period in any of the animal groups.

### Full blood count

Differences in full blood counts between the groups are shown in Table 1. There was a fall in both total white cell and platelet counts in all the CLP groups in comparison with the no surgery and sham groups. The CLP and testosterone administered group had the greatest fall in platelet count which was significantly less than both the CLP alone and the CLP and danazol groups.

### Total protein

There was a significant fall in total protein values in all the CLP groups, irrespective of testosterone or danazol treatment, in comparison with the no surgery or sham groups (Table 2).

### Measurement of acute phase proteins

Figure 1 illustrates the typical changes observed in the serum electrophoresis profiles of a control rat and a rat following CLP. A fall in albumin levels occurred in all CLP groups in comparison with the no surgery and sham groups. As shown in Table 2, the $\alpha_1$ proteins fell in the CLP alone and CLP and testosterone groups in comparison with the control groups.

**Table 1**. Full blood count results in the different animal groups at the end of the study period

| | No surgery (n = 6) | Sham (n = 9) | CLP alone (n = 9) | CLP + Test (n = 12) | CLP + Dan (n = 12) |
|---|---|---|---|---|---|
| White cell count ($\times 10^9$/l) | $7.9 \pm 0.4$ | $7.14 \pm 0.8$ | $4.8 \pm 0.59^{*}$ | $5.3 \pm 0.5^{*}$ | $6.3 \pm 0.8$ |
| Platelets ($\times 10^9$/l) | $959 \pm 23$ | $848 \pm 34$ | $697 \pm 39^{**}$ | $445 \pm 41^{**,\dagger,\dagger\dagger}$ | $654 \pm 47^{**}$ |
| Neutrophils (%) | $18.8 \pm 1.7$ | $16.14 \pm 3.4$ | $14.7 \pm 1.9$ | $19.2 \pm 1.8$ | $19.7 \pm 4.2$ |
| Lymphocytes (%) | $80 \pm 1.4$ | $82 \pm 3.5$ | $79 \pm 2.6$ | $79.3 \pm 1.8$ | $78.5 \pm 4.3$ |
| Monocytes (%) | $1.8 \pm 0.2$ | $1.8 \pm 0.3$ | $2 \pm 1$ | $1.5 \pm 0.2$ | $1.6 \pm 0.4$ |

* $P < 0.05$ from No surgery and Sham groups; ** $P < 0.01$ from No surgery and Sham; † $P < 0.01$ from CLP alone; †† $P < 0.01$ from CLP + Dan.

**Table 2**. Total protein, albumin and the $\alpha_1$, $\alpha_2$, $\beta_1$, $\beta_2$ and $\gamma$ globulin fractions on electrophoresis in the different animal groups

| | No surgery (n = 6) | Sham (n = 9) | CLP alone (n = 9) | CLP + Test (n = 12) | CLP + Dan (n = 12) |
|---|---|---|---|---|---|
| Total protein (g/l) | $54.7 \pm 2.3$ | $50.5 \pm 1.7$ | $43.4 \pm 3.2^{**}$ | $39.5 \pm 2.3^{**}$ | $43.5 \pm 1.8^{*,\dagger}$ |
| Albumin (g/l) | $27.7 \pm 1.2$ | $22.7 \pm 1.4$ | $13.8 \pm 1.4^{***}$ | $15.5 \pm 1.2^{***}$ | $14.8 \pm 0.5^{***}$ |
| $\alpha_1$ (g/l) | $9.6 \pm 0.6$ | $8.8 \pm 0.5$ | $5.9 \pm 0.7^{*}$ | $8.1 \pm 0.8$ | $10.1 \pm 1.1^{\dagger\dagger}$ |
| $\alpha_2$ (g/l) | $4.1 \pm 0.4$ | $5.4 \pm 0.4$ | $4.3 \pm 0.5$ | $5.5 \pm 0.5$ | $5.6 \pm 0.6$ |
| $\beta_1$ (g/l) | $2.3 \pm 0.2$ | $2.8 \pm 0.3$ | $2.1 \pm 0.5$ | $1.8 \pm 0.2$ | $3.4 \pm 0.5^{\S}$ |
| $\beta_2$ (g/l) | $7.1 \pm 0.4$ | $7.5 \pm 0.5$ | $5.8 \pm 0.8$ | $6.5 \pm 0.4$ | $6.9 \pm 0.3$ |
| $\gamma$ (g/l) | $2.5 \pm 0.4$ | $2.9 \pm 0.4$ | $2.4 \pm 0.7$ | $1.9 \pm 0.3$ | $2.4 \pm 0.6$ |
| Complement $C_3$ (g/l) | $0.41 \pm 0.03$ | $0.42 \pm 0.04$ | $0.38 \pm 0.04$ | $0.32 \pm 0.03$ | $0.36 \pm 0.03$ |
| Transferrin (g/l) | $0.62 \pm 0.01$ | $0.78 \pm 0.04$ | $0.74 \pm 0.04$ | $0.7 \pm 0.03$ | $0.7 \pm 0.03$ |

* $P < 0.05$, ** $P < 0.01$, *** $P < 0.001$ from No surgery and Sham groups; † $P < 0.05$, †† $P < 0.01$ from CLP alone; § $P < 0.05$ from CLP + Test.

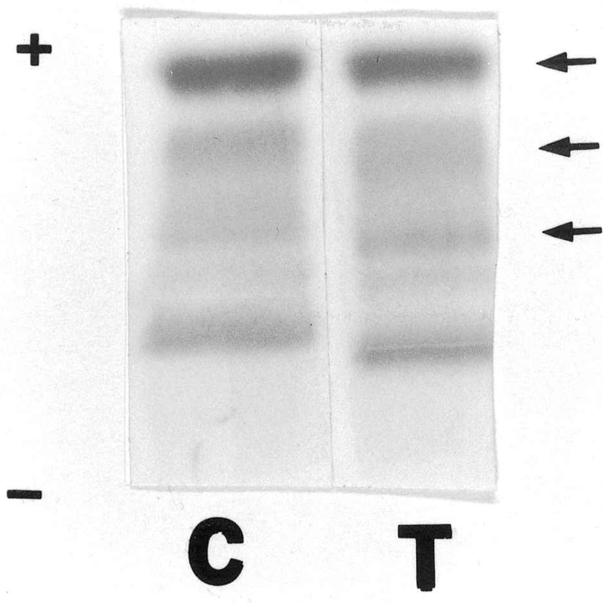

FIG. 1. Electrophoresis strips from a normal rat (C) and a rat following CLP (T) are shown. The arrows indicate in sequence from above down, diminished albumin, a decrease in the $\alpha_1$ and an increase in the $\alpha_2$ bands and an increase in the $\beta_1$ band respectively.

This fall was reversed in the CLP and danazol group in which levels of the $\alpha_1$ protein fraction were significantly greater than the CLP alone group. Danazol administration also tended to increase the $\alpha_2$, $\beta_1$ and $\beta_2$ proteins in comparison with the CLP alone and CLP and testosterone groups. There was no change in the $\gamma$ protein fraction or the complement $C_3$ levels between any of the groups. Transferrin levels tended to rise in the sham group and all of the CLP groups in comparison with the no surgery group, although these differences were not significant.

### Discussion

In this model of caecal ligation and puncture, surgery and sepsis induces a profound change in the plasma protein profiles of the rat (Fig. 1). When these results are analysed qualitatively it is clear that danazol, a derivative of the synthetic steroid 17-ethynl testosterone increases plasma acute phase proteins as measured by increased $\alpha_1$, $\alpha_2$ and $\beta_1$ protein fractions on serum electrophoresis. Similar but less impressive trends were also observed with testosterone proprionate.

The levels of $\alpha_1$ inhibitor 3 ($\alpha_1$I3), the major negative acute phase protein in the rat, was maintained in this study by danazol. In contrast, in the CLP alone and the CLP and testosterone groups $\alpha_1$ levels fell, implying that danazol either inhibited the fall in this negative acute phase protein or increased other positive acute phase proteins in this band. In addition, these results show that danazol also increased the $\alpha_2$ band on electrophoresis suggesting that $\alpha_2$-macroglobulin levels, the major rat positive acute phase protein and contained in this band, was also increased. While serum electrophoresis gives a useful indication of serum protein changes induced by treatment in these studies, characterization of individual acute phase proteins require further study.

Little information is available regarding the clinical importance of appropriate acute phase responses to inflammation. However, the highly conserved nature of the major acute phase proteins implies a significant survival advantage. Furthermore, many of the acute phase proteins are protease inhibitors and are presumed to be essential in modifying the major life-threatening side effects of sepsis and tissue trauma. Dominioni and colleagues have demonstrated that acute phase responses are indicative of outcome in severe sepsis.[3] In a series of 135 patients with surgical infections, patients with low $\alpha$–1-acid glycoprotein, $\alpha$–1-antitrypsin, complement factor B and $C_3$ levels at presentation had a higher mortality.

Recombinant protease inhibitors can be used effectively in the treatment of sepsis. Guerrero and co-workers have shown that administration of C1 esterase inhibitor prevents the pulmonary and cardiovascular dysfunction associated with endotoxic shock.[6] Dickneite and Seiffge have demonstrated that C1 esterase inhibitor prevents the vascular leak syndrome in the systemic inflammatory response syndrome (SIRS) induced in rats by interleukin-2.[7] In humans, Hack and colleagues have also shown that C1-esterase inhibitor administration appear to be of benefit in septic patients.[8] These studies provide evidence that exogenous administration of acute phase proteins might, therefore, be of benefit in patients with sepsis.

An alternative approach arises from our work demonstrating the induction of acute phase proteins using danazol. Danazol is known to induce C1-esterase levels and is used clinically in angiooedema, a C1-esterase inhibitor deficient state.[9] Endogenous induction of C1-esterase inhibitor levels using danazol would have both medical and economic advantages. Furthermore, our work demonstrates that anabolic steroids may offer the potential in the prophylactic treatment of subjects at increased risk of sepsis. The effectiveness of danazol in inducing increased acute phase protein levels and altering physiological and clinical parameters in sepsis, therefore, warrants further evaluation.

### References

1. Kushner I. The phenomenon of the acute phase response. *Ann New York Acad Sci* 1992; **389**: 39–48.
2. Fey G, Gaudie J. The acute phase response of the liver in inflammation. In: Popper H, Schaffner F, eds. *Progress in Liver Disease*. Philadelphia: W. B. Saunders, 1990.
3. Dominioni L, Dionigri R, Zanello M, Monico R, Cremaschi R, Ballabio A, Massa M, Cornelli M, Dal Ri P, Pisati P. Sepsis score and acute phase protein response as predictors of outcome in septic surgical patients. *Arch Surg* 1987; **122**: 141–146.
4. Fourrier F, Chopin C, Huart J-J, Runge I, Caron C, Gondemand J. Double-blind, placebo controlled trial of antithrombin III concentrate in septic shock with disseminated intravascular coagulation. *Chest* 1993; **104**: 882–888.
5. Redens TB, Emerson TE. Antithrombin III treatment limits disseminated intravascular coagulation in endotoxemia. *Circ Shock* 1989; **28**: 49–58.
6. Guerrero R, Velasco F, Rodriguez M, Lopez A, Rojas R, Alvarez M, Villalh R, Rubio V, Torres A, del Castillo D. Endotoxin-induced pulmonary dysfunction is prevented by C1-esterase inhibitor. *J Clin Invest* 1993; **91**: 2754–2760.
7. Dickneite G, Seiffge D. Efficacy of C1-inhibitor on capillary leakage and septic shock in the rat. In: Faist E, Baue AE, Schildberg FW, eds. *The Immune Consequences of Trauma, Shock and Sepsis—Mechanisms and Therapeutic Approaches Vol. 1*. Lengerich: PABST Science Publishers, 1996.
8. Hack CR, Voerman HJ, Eisele B, Hack C, Voerman H, Eisele B, Keinecke H-O, Nuijens J, Eerenberg A, Ogilivie A, van Schijndel S, Delvos V, Thijs L. C1-esterase inhibitor substitution in sepsis. *Lancet* 1992; **339**: 378.
9. Gelfand JA, Frank MM. Treatment of hereditary angioedema with danazol: reversal of clinical and biochemical abnormalities. *New Engl J Med* 1976; **295**: 1444–1448.

ACKNOWLEDGEMENT. This work was supported by the Royal City of Dublin Hospital Trust.

**Received 11 November 1996;
accepted in revised form 9 December 1996**
